# Community-based analysis of stroke prevention and effect of public interventions in atrial fibrillation: results from the ARENA project

**DOI:** 10.1007/s00392-024-02510-6

**Published:** 2024-08-08

**Authors:** Maura M. Zylla, Belgin Özdemir, Matthias Hochadel, U. Zeymer, Ibrahim Akin, Armin Grau, Steffen Schneider, Angelika Alonso, Bernd Waldecker, Tim Süselbeck, Harald Schwacke, Markus Haass, Ralf Zahn, Martin Borggrefe, Jochen Senges, Norbert Frey, Dierk Thomas

**Affiliations:** 1 Department of Cardiology, University of Heidelberg, Medical University Hospital, Im Neuenheimer Feld 410, 69120 Heidelberg, Germany; 2 HCR (Heidelberg Center for Heart Rhythm Disorders), Medical University Hospital Heidelberg, Im Neuenheimer Feld 410, 69120 Heidelberg, Germany; 3 DZHK (German Centre for Cardiovascular Research), partner site Heidelberg/Mannheim, 69120 Heidelberg, Germany; 4 Stiftung Institut für Herzinfarktforschung, IHF, Bremserstraße 79, 67063 Ludwigshafen, Germany; 5 Department of Cardiology, Hospital Ludwigshafen, Bremserstraße 79, 67063 Ludwigshafen, Germany; 6 Department of Cardiology, Angiology and Pneumology, I. Medizinische Klinik, University Medical Center Mannheim, Theodor-Kutzer-Ufer 1-3, 68167 Mannheim, Germany; 7 Department of Neurology, Hospital Ludwigshafen, Bremserstraße 79, 67063 Ludwigshafen, Germany; 8 Department of Neurology, University Medical Center Mannheim, Theodor-Kutzer-Ufer 1-3, 68167 Mannheim, Germany; 9 Department of Cardiology and Angiology, GRN Klinikum Schwetzingen, Bodelschwinghstr. 10, 68723 Schwetzingen, Germany; 10 Kardiologische Praxisklinik, 67071 Ludwigshafen, Germany; 11 Diakonissen-Stiftungs-Krankenhaus Speyer, 67346 Speyer, Germany; 12 Theresienkrankenhaus Mannheim, Bassermannstraße 1, 68165 Mannheim, Germany

**Keywords:** Atrial fibrillation, Community-based intervention, Oral anticoagulation, Patient-reported outcomes, Quality of life

## Abstract

**Background:**

Community-based interventions may promote awareness and adherence to atrial fibrillation (AF)-related therapies, potentially reducing adverse events. The ARENA project investigated the health status, therapies and events in AF patients in the Rhein–Neckar Region, Germany. The subproject “ARENA intervention” studied the effect of community-based interventions on AF-associated outcomes.

**Methods:**

From 2016 onward, patients with diagnosed AF were recruited for the observational ARENA registry. In 2018, an intervention period was initiated involving population-based information campaigns on AF diagnosis and therapies. The “control group” was recruited prior to initiation, and the “intervention group” afterward. Patients underwent standardized follow-up > 1 year after recruitment. Clinical outcomes, therapy and quality of life were compared between the two groups.

**Results:**

A total of 2769 patients were included. This real-world cohort showed high adherence to oral anticoagulation therapy (OAC) and an increased use of NOACs over vitamin K antagonists over time. In the intervention group (*n* = 1362), more patients continued OAC at follow-up (87.1% vs. 81.5%, *P* = 0.002). However, this difference was not significant in the patient subgroup with class I/IIa indications for OAC (90.1% vs. 87.5%, *P* = 0.11). AF-related re-hospitalization was lower in the intervention group (6.8% vs. 12.3%, *P* < 0.001). There was no significant difference in quality of life. AF-related anxiety was reduced at follow-up. Of note, nearly a quarter of all patients stated that ARENA had influenced their health perception.

**Conclusion:**

Tailored community-based campaigns may raise awareness for AF-related health issues, supporting therapy adherence. Future public strategies to improve quality of life in AF patients should be investigated, as the ARENA project hints at a potential benefit of population-based campaigns.

**Trial registration:**

ClinicalTrials.gov (Identifier: NCT02978248).

**Supplementary Information:**

The online version contains supplementary material available at 10.1007/s00392-024-02510-6.

## Introduction

Atrial fibrillation (AF) constitutes a major health burden due to its continuously increasing prevalence, as well as its associated risks and complications. AF-related symptoms and thromboembolic complications are a frequent cause of emergency service contacts, hospitalizations, and overall morbidity in our society. In contrast to other common diseases, the general awareness in the population for AF and its implications is low. [1, 2] With respect to other cardiac conditions, e.g. hypertension or cardiovascular disease, community-based interventions have been efficient in bringing the importance of early diagnosis and therapy into public attention. [3–5] In the context of AF, community-based interventions may promote awareness of typical symptoms, facilitate earlier diagnosis, and improve adherence to recommended therapeutic measures and medication. As a consequence, appropriate tailored initiatives may lead to a reduction in AF-related adverse events and an improvement in quality of life at a community level.

The Rhein–Neckar Region in southern Germany is populated by more than two million inhabitants. It contains both rural and urban environments reflecting a balanced population structure with respect to age and ethnicity. Local health-care providers include more than 40 hospitals, > 300 medical practices, and > 300 pharmacies which are involved in the medical care of AF patients. The ARENA registry sets out to investigate the health status, quality of life, as well as concomitant medical and interventional therapeutic strategies in patients with AF in the Rhein–Neckar Region. [6] The subproject “ARENA intervention” studied the effects of community-based interventions on AF-associated outcomes with a focus on stroke prevention.

## Methods

### The ARENA registry

The prospective, observational ARENA project was initiated in 2016 by the “Institut für Herzinfarktforschung” (IHF) and established in collaboration with the cardiology and neurology departments of local tertiary care hospitals (Heidelberg University Hospital, University Medical Center Mannheim; Hospital Ludwigshafen), as well as health-care providers of the private practice sector and pharmacies in the Rhein–Neckar Region in southern Germany. At recruitment and during the course of the study, the health status, AF-related pharmacological and interventional therapy, quality of life, and long-term clinical outcome parameters in a real-world cohort of AF patients were examined. The study was conducted according to the principles of good clinical practice, approved by local ethics committees (Landesärztekammer Rheinland-Pfalz, registration number 837.366.15 (10,134) from 30th March 2016) and registered at ClinicalTrials.gov (Identifier: NCT02978248).

The subproject “ARENA intervention” aimed at investigating the effect of community-based measures on health status, therapy adherence, and outcome in patients with AF. The intervention period was initiated in April 2018 involving an extensive population-based campaign promoting information on diagnosis, associated risks, and therapies in AF. The campaign included different forms of initiatives, including broadcast in local media (5 television broadcasts, 4 radio interviews), 41 postings on social media channels, 29 public seminars, presentations during local trade fairs, exhibitions and congresses, distribution of print information, 28 published articles in print media, and AF screening events using the portable detection device (“MyDiagnostick”, Applied Biomedical Systems, NL) in 36 pharmacies and public events organized by the IHF. Of the 5149 patients screened with the respective device, a positive result indicating the potential presence of AF was obtained in 409 individuals. Additionally, a local self-help group for patients with AF was established.

### Recruitment and study cohorts

All local health-care providers for AF patients in the Rhein–Neckar Region were invited to participate in this registry. Patients ≥ 18 years in whom AF had been diagnosed were recruited at presentation at the respective local health-care center or in the context of public outreach campaigns. Sixteen medical centers (11 hospitals and 5 medical practices) participated in patient recruitment.

The overall cohort was recruited in the period from 08/2016 to 12/2018 and comprised two subgroups: the “control group” was recruited before the start of the widespread community-based information campaign in 04/2018, and the “intervention group” was recruited from this time until 01/2019. Patients provided written informed consent for participation in the registry and for statistical processing of their pseudonymized data.

The majority of patients were recruited either as inpatients during hospitalization events (55.5%) or during outpatient visits (20.7%), which were AF related in 55.2% of cases and in 42.6% due to other cardiovascular conditions. Other study recruitment strategies consisted in postal written contact (19.5%) or screening as part of a public outreach campaign (4.3%). In the “control group”, more patients were recruited during in-hospital stay (70.3%), whereas recruitment via postal contact and during hospitalization was similarly common in the “intervention group” (39.6% and 40.2%).

### Data collection and follow-up

Patient characteristics, medical history, and pharmaceutical and non-pharmaceutical therapy were systematically enquired at the time of recruitment from the recruiting center and the patients. Additionally, participants were provided with structured baseline questionnaires regarding quality of life at 2–4 weeks after baseline visit, or together with the written letter in case of postal recruitment. For the assessment of stroke and bleeding risk as well as the indication for OAC, CHA_2_DS_2_VASc and HAS-BLED scores were calculated from the patient status at baseline. [7] Data were recorded in a central database, coordinated by the IHF.

Patients underwent standardized telephone and postal contact based follow-up interrogations coordinated by the IHF scheduled at > 1 year after recruitment. Additional follow-up visits could be recorded and transmitted to the IHF by their physicians at any time point of medical contact on a voluntary basis. In case of stroke, severe bleeding, and peripheral embolism leading to hospitalization, associated medical documentation was procured from the respective medical center. Endpoint data were procured from patients or their relatives, medical charts of participating centers, and from local registration offices to assess the effects of the intervention. Information available from local registration offices was restricted to the vital status of the participant.

Quality of life was measured by the EQ-5D-5L and additional pre-specified questions aiming at the psychosocial status, health perception, and anxiety. The item values from the EQ-5D-5L-categories were converted to an index score using the German value set as a reference. [8] The index values range from -0.661 to 1.0, with a score of 1.0 reflecting the best possible subjective health perception. Results from the visual analog scale (VAS) with a value range of 0 (worst perceived health status) to 100 (best perceived health status) are presented separately.

To describe how many patients followed a rhythm control concept, we summarized those patients in whom either current antiarrhythmic medical therapy, previous cardioversion, or catheter ablation was documented.

### Statistical analysis

Descriptive analysis of baseline and outcome parameters was performed for the overall cohort and each of the two subgroups, respectively. The “control group” and “intervention group” were defined by recruitment before or from 19 April 2018 onward, respectively. Groups were compared with respect to clinical course, therapy, and quality of life to analyze the effect of community-based campaigns. An additional subgroup analysis was performed for patients with available CHA_2_DS_2_VASc score and with a long-term class I or IIa indication of oral anticoagulation therapy (OAC) according to current guidelines. For this evaluation, patients who previously had received an LAA occluder or with temporary indication for OAC (men with a CHA_2_DS_2_VASc score of 0 and women with a CHA_2_DS_2_VASc score of 1 undergoing cardioversion or AF ablation) were excluded.

Continuous data are reported as median (with inter-quartile range; Q1 = 25th percentile, Q3 = 75th percentile) or mean ± standard deviation (SD), and categorical data are presented as patient counts and percentages. The Mann–Whitney–Wilcoxon test was applied for between-group comparisons of ordinal or metric variables, and the Pearson Chi-square test for binary variables, or Fisher’s exact test in case of infrequent events. One-year mortality at 365 days after enrollment was calculated using the Kaplan–Meier estimator with confidence intervals computed via log–log transformation, and compared by log-rank test. One-year rates of non-fatal follow-up events with documented date are reported as rates among survivors and restricted to 365 days after enrollment. Information concerning the patient status, such as symptoms, current medication, or quality of life, was known at the time of last long-term follow-up contact. For comparison of baseline and follow-up medication in the respective subgroups, paired analyses were conducted. To account for correlations between FU and baseline medication, the confidence intervals for the risk differences and the interaction p values were calculated using generalized estimating equations for binary data with identity link function.

The descriptive parameters were calculated from the available cases and the number of patients with available data indicated in the respective figures and tables. Reasons for incomplete follow-up are given in “Results”. All statistical comparisons were two sided. *P* values ≤ 0.05 were considered statistically significant. Adjustment for multiple testing was not performed. The statistical analysis was performed at the biometrics department of the IHF (Ludwigshafen) using SAS 9.4 software (SAS Institute, Cary, NC, USA).

## Results

### Characteristics of the ARENA patient cohort

In total, 2,769 patients were recruited in the ARENA registry. Mean age was 72.5 ± 10.9 years and 62.6% of participants were male (Table 1). The majority of patients were diagnosed with paroxysmal AF (Table 1). A concomitant cardiac condition was present in 76.6% of patients. Hypertension was the most the most common cardiac co-morbidity, followed by coronary artery disease (Table 1). Median left ventricular ejection fraction (LVEF) was preserved in the majority of cases (Table 1). Mean left atrial diameter was 45.0 ± 7.2 mm. Additional cardiovascular risk factors were common, in particular arterial hypertension, diabetes, and renal failure (Table 1). Mean CHA_2_DS_2_-VASc score was 3.6 ± 1.7 and mean HAS-BLED score was 2.1 ± 1.1. Prior stroke or transient ischemic attack (TIA) had been diagnosed in 13.7% of patients. Anticoagulation therapy was prescribed in 88.4% of cases at baseline, using NOACs in the majority of cases (66.9%). In 20.0% of participants vitamin K antagonists (VKA) were prescribed. Antiplatelet agents were present in 17.0% of patients at recruitment, and in 11.0% as a combination therapy in either a dual combination or triple combination scheme with OAC therapy. Rivaroxaban (26.3%) and apixaban (25.1%) were the most frequently employed NOACs at baseline in the overall cohort, followed by dabigatran (11.9%) and edoxaban (3.7%). Beta-blockers were prescribed in 75.4% of cases, and digitalis in 11.3%. A rhythm control strategy with cardioversion, antiarrhythmic medical therapy, or catheter ablation was chosen or had been attempted in 1016 (42.7%) patients, whereas rate control was documented as the primary strategy in 1339 (56.3%) patients. Patients in whom a rhythm control concept was followed were younger (71.5 ± 9.6 years vs. 75.5 ± 9.2 years, *P* < 0.001), less often affected by some cardiac co-morbidities (coronary artery disease: 37.6% vs. 45.8%, *P* < 0.001; dilated cardiomyopathy: 11.0% vs. 8.1%, *P* = 0.015), and less often had a history of stroke or TIA (11.0% vs. 17.4%, *P* < 0.001). We cannot exclude that some patients in whom rhythm control therapies were documented had later been switched to rate control, e.g., due to previous unsuccessful rhythm control attempts. However, patient characteristics in the subgroups of rate or rhythm control are consistent with those of patients predominantly assigned to the respective therapeutic strategy in everyday clinical practice. Pharmacological antiarrhythmic therapy was present in 3.6% of patients with class I antiarrhythmic drugs and in 6.5% with class III antiarrhythmic agents. Nearly one in five patients had undergone catheter ablation of AF and nearly a third of the cohort had undergone cardioversion (Table 1). The majority of patients described absent (43.5% EHRA I) or mild (43.1% EHRA II) AF-associated symptoms. Shortness of breath during everyday activity was present in the majority of cases (36.5% NYHA II; 26.2% NYHA III/IV). The quality of life reflected in the EQ-5D-5L score was 0.89 [Q1: 0.77; Q3: 0.97]. Subjectively perceived health status was mildly reduced according to VAS score (70 [Q1: 50; Q3: 80]) at recruitment.

**Table 1:** ARENA cohort patient characteristics at recruitment

	All patients (*N* = 2769)	Control group (*N* = 1407)	Intervention group (*N* = 1362)	*P* value
Age (years, mean ± SD)	72.5 ± 10.9 (*N* = 2690)	71.4 ± 11.4 (*N* = 1399)	73.7 ± 10.3 (*N* = 1291)	< 0.001
Female (%)	37.4 (1026/2742)	37.9 (531/1402)	36.9 (495/1340)	0.61
Paroxysmal AF (%)	60.6 (1438/2374)	56.9 (781/1372)	65.6 (657/1002)	< 0.001
Persistent AF/long-standing persistent AF (%)	39.4 (936/2374)	43.0 (590/1372)	34.5 (345/1002)	< 0.001
First diagnosis of AF (%)	13.7 (360/2631)	16.9 (234/1385)	10.1 (126/1246)	< 0.001
CHA_2_DS_2_-VASc score (mean ± SD)	3.6 ± 1.7 (2527)	3.4 ± 1.8 (1360)	3.8 ± 1.7 (1167)	< 0.001
CHA_2_DS_2_-VASc score > 2, (%)	86.5 (2187/2527)	83.9 (1141/1360)	89.6 (1046/1167)	< 0.001
HAS-BLED score	2.1 ± 1.1 (2489)	2.0 ± 1.1 (1360)	2.3 ± 1.1 (1129)	< 0.001
EHRA III/IV	13.4 (332/2479)	15.3 (206/1345)	11.1 (126/1134)	0.002
Previous catheter ablation of AF	19.1 (505/2650)	18.9 (261/1384)	19.3 (244/1266)	0.79
Previous cardioversion	31.3 (830/2651)	34.5 (478/1384)	27.8 (352/1267)	< 0.001
Cardiac co-morbidities (%)	76.6 (2011/2624)	74.8 (1025/1371)	78.7 (986/1253)	0.018
Coronary artery disease	39.3 (1031/2624)	37.2 (510/1371)	41.6 (521/1253)	0.022
Heart failure (%)	22.1 (579/2624)	19.7 (270/1371)	24.7 (309/1253)	0.002
Left ventricular ejection fraction (LVEF) (%,median [Q1;Q3])	51.8 ± 13.0 (*N* = 1511)	51.6 ± 13.1 (*N* = 1006)	52.1 ± 12.9 (*N* = 505)	0.65
Previous PM/ICD/CRT implantation	24.6 (651/2648)	29.9 (414/1384)	18.8 (237/1264)	< 0.001
Diabetes mellitus	25.1 (673/2685)	24.5 (337/1376)	25.7 (336/1309)	0.48
Renal failure (%)	19.6 (525/2685)	18.3 (252/1376)	20.9 (273/1309)	0.097
GFR (MDRD)	66.6 ± 26.5 (*N* = 1639)	68.7 ± 26.5 (*N* = 1103)	60.9 ± 25.6 (*N* = 536)	< 0.001
Hypertension	74.1 (1990/2685)	75.7 (1041/1376)	72.5 (949/1309)	0.062
Smoker	7.3 (192/2625)	7.4 (102/1376)	7.2 (90/1249)	0.84
Previous stroke/TIA	13.7 (367/2685)	11.3 (144/1376)	16.2 (212/1309)	< 0.001
Previous relevant bleeding complication	4.4 (90/2048)	3.5 (48/1375)	6.2 (42/673)	0.004

*AF* atrial fibrillation, *CRT* cardiac resynchronization therapy, *EHRA* European Heart Rhythm Association, *GFR* glomerular filtration rate, *ICD* implantable cardioverter-defibrillator, *LVEF* left ventricular ejection fraction, *PM* pacemaker, *SD* standard deviation, *TIA* transient ischemic attack

### Baseline characteristics and therapy in subgroups of “ARENA Intervention”

**Fig. 1:**
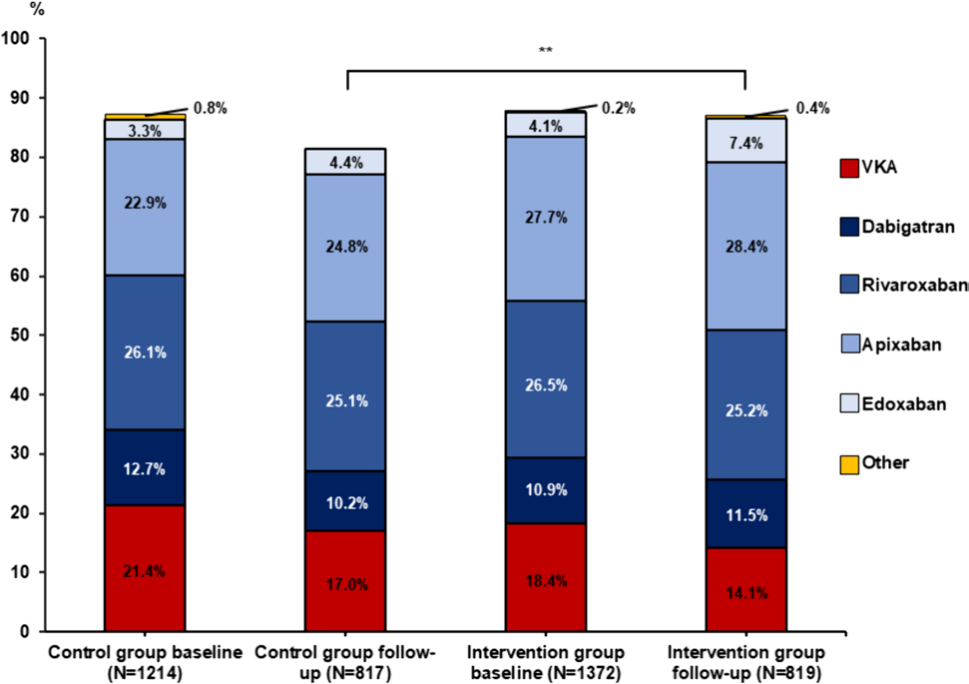
Oral anticoagulation in the control vs. intervention group. NOACs constituted the preferred agent for OAC therapy in both subgroups with a decline in use of vitamin K antagonists over the course of the observation period. Other therapies for stroke prevention include subcutaneous heparin administration, which was applied in a minority of cases. Rates of OAC prescription did not differ significantly between the subgroups at baseline (*P* = 0.63). At follow-up, there was a higher adherence to OAC-therapy in the intervention group in comparison to the control group. *VKA* vitamin k antagonist ** = *P* < 0.01

Patients recruited during the intervention period were older than patients in the control period and were more often affected by cardiac co-morbidities (Table 1). The most structural cardiac conditions conditions were coronary artery disease and heart failure. However, mean left ventricular systolic function was not statistically different between groups in cases with documented echocardiography at baseline (Table 1). Sex distribution was similar in both groups. More patients in the intervention period were diagnosed with paroxysmal AF which was the most common type of AF in both groups. First diagnosis of AF was more common in the control period (Table 1). There was no statistically significant difference in common cardiac risk factors such as smoking, hypertension, diabetes mellitus, or renal failure. However, median glomerular filtration rate was slightly lower in the intervention group (Table 1). Additionally, more patients in the intervention group had a history of cerebral thromboembolic complications (Table 1). The mean CHA_2_DS_2_-VASc and HAS-BLED scores in the intervention group were higher (Table 1). A CHA_2_DS_2_-VASc score of ≥ 2 was recorded in 89.5% of patients in the intervention group and 83.9% of patients in the control group (*P* < 0.001). AF-related symptoms were more pronounced in the control group (Table 1). The quality of life at baseline as reflected in the EQ-5D-5L-index 2 weeks after recruitment was not different between the two groups (control group: 0.89 [Q1 = 0.77; Q3 = 0.96]; intervention group: 0.89 [Q1 = 0.78; Q3 = 0.97]). However, with respect to specific interrogation dedicated to anxiety or cardiophobia, more patients in the control period stated that they perceived nervousness, fear of acute cardiac events, and avoided activity leading to increase in heart rate, whereas patients in the intervention group were less often anxious with respect to heart-related symptoms or physical activity (Supplemental Table 1). More patients in the intervention phase had undergone medical or pharmacological cardioversion or device implantation, whereas there was no significant difference in the rates of previous AF ablation (Table 1). At baseline, there was no statistically significant difference in oral anticoagulation rates (Table 2 and Fig. 1). More patients received NOACs in the intervention group (Table 2 and Fig. 1). Apixaban was prescribed more often in the intervention group (control group: 22.9%, intervention group: 27.7%, *P* = 0.004, Fig. 1). Interventional therapies for stroke prevention by LAA occluder were not statistically different between groups (control period: 0.6%, intervention period: 0.7%, *P* = 0.62) at baseline. Concomitant antiplatelet therapy was prescribed in a minority of patients in both groups (Table 2). At baseline, beta-blockers and antiarrhythmic medication were more often prescribed in the control group (Table 2).

**Table 2:** Pharmacological therapy in ARENA subgroups at baseline and follow-up

	Control group (paired data)			Intervention group (paired data)			*P*-value follow-up intervention vs. control	*P*-value interaction*
	Baseline	Follow-up	RD (95%-CI)* follow-up vs. baseline	Baseline	Follow-up	RD (95%-CI)* follow-up vs. baseline		
Anticoagulation (%, *N*)	88.5 (709/801)	81.8 (655/801)	−6.7 (−9.2 —−4.3)	89.3 (658/737)	87.0 (641/737)	−2.3 (−4.4—−0.2)	0.005	0.007
NOAC (%,* N*)	66.8 (532/797)	64.6 (515/797)	−2.1 (−5.0— + 0.7)	69.5 (505/727)	72.6 (528/727)	+ 3.2 (+ 0.9— + 5.4)	< 0.001	0.005
VKA (%, *N*)	20.2 (161/797)	17.1 (136/797)	−3.1 (−4.8—−1.4)	19.3 (140/727)	14.0 (102/727)	−5.2 (−7.0—−3.4)	0.10	0.098
Antiplatelet agents (%, *N*)	13.2 (105/794)	6.3 (50/794)	−6.9 (−9.3—−4.6)	15.5 (98/632)	8.4 (53/632)	−7.1 (−9.7—−4.6)	0.13	0.91
In combination with OAC (%, *N*)	8.8 (69/787)	2.2 (17/787)	−6.6 (−8.6—−4.6)	10.1 (63/624)	2.9 (18/624)	−7.2 (−9.6—−4.9)	0.38	0.70
Beta-blocker (%, *N*)	76.4 (604/791)	74.2 (587/791)	−2.1 (−5.3— + 1.0)	72.7 (460/633)	70.3 (445/633)	−2.4 (−5.7— + 0.9)	0.10	0.92
Digitalis (%, *N*)	9.4 (74/791)	11.4 (90/791)	+ 2.0 (−0.2— + 4.2)	10.3 (65/633)	12.2 (77/633)	+ 1.9 (−0.5— + 4.2)	0.65	0.94
AAD (%, *N*)	12.7 (98/773)	10.6 (82/773)	−2.1 (−4.5— + 0.4)	8.7 (51/584)	7.5 (44/584)	−1.2 (−3.3— + 0.9)	0.053	0.60

*In order to account for correlations between FU and baseline medication, the confidence intervals for the risk differences and the interaction *p*-values have been calculated using Generalized Estimating Equations for binary data with identity link function

*AAD* antiarrhythmic drugs, *NOAC* novel oral anticoagulant, *OAC* oral anticoagulation, *VKA* vitamin K antagonist

### Clinical long-term follow-up

Follow-up data was procured until at a median of 665 days [Q1: 568 days; Q3: 1041 days] after recruitment in 93.9% of baseline survivors. In 16.5%, only information on vital status was available from local registration offices. In the control group, follow-up data on 1282 patients (93.7%) were available after 706 days [Q1: 516 days; Q3:1176 days] and in 1266 (94.1%) participants in the intervention group with a median follow-up duration of 630 days [Q1: 605 days; Q3: 995 days]. Apart from vital status, further information on patient status and clinical events was available in 61.9% of patients of the overall cohort. In patients lost to follow-up, reasons for unavailability of further clinical end points included death (31.8%), withdrawal of consent for further follow-up interrogations (24.6%), failure to contact the patient and information only available from the local registration office (23.5%), information only available from relatives of the patients (3.4%), or other personal reasons for non-response (16.8%) without significant difference between the groups (loss to follow-up for additional end points control group: 38.3%; intervention group: 37.9%, *P* = 0.84).

**Fig. 2:**
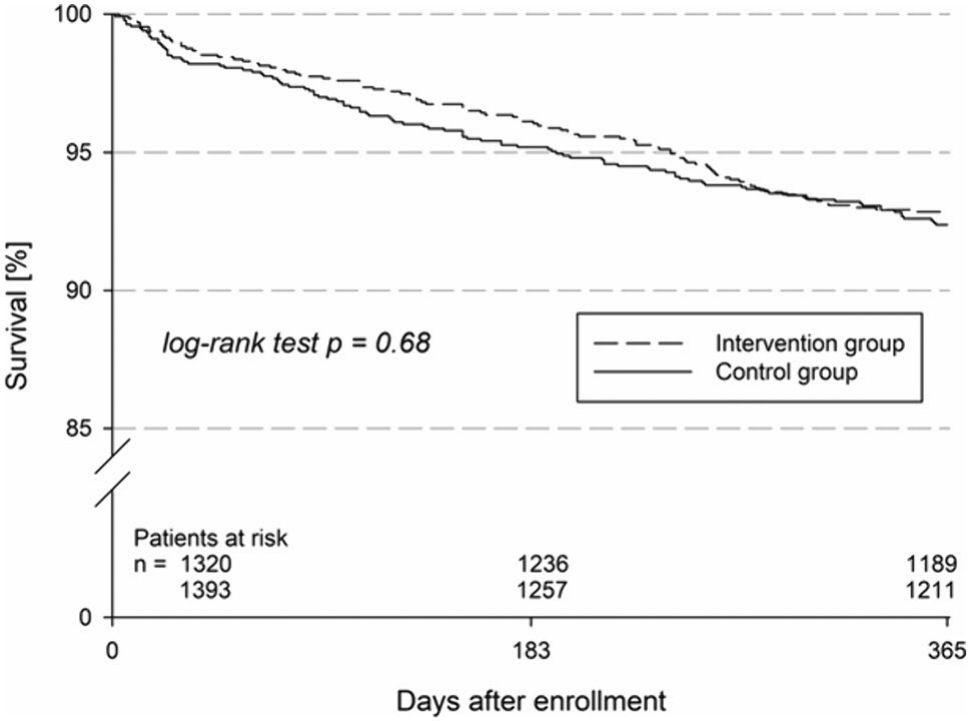
One-year survival in the control vs. intervention group. Kaplan–Meier analysis of all-cause mortality. There was no statistically significant difference between the two groups

Estimated 1-year-mortality rate was 7.6% (CI 6.3–9.2%) in the control group and 7.2% (CI 5.9–8.8%) in the intervention group (*P* = 0.68, Fig. 2). Kaplan–Meier estimates of combined end points of death, myocardial infraction, stroke (MACCE) (control group: 8.2% [CI 6.8–9.8%]; intervention group 7.9% [CI 6.6–9.5%], *P* = 0.81) and death, myocardial infarction, stroke, and major bleeding (control group: 8.5% [CI 7.1–10.2%]; intervention group 8.5% [CI 7.1–10.1%], *P* = 0.92) were not statistically different between groups.

**Fig. 3:**
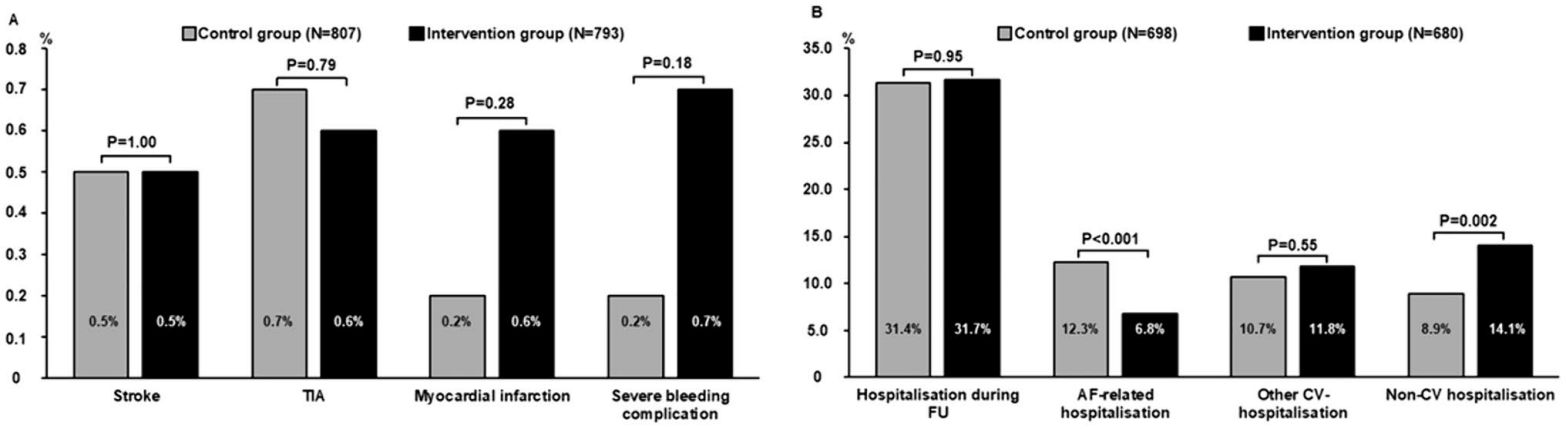
Clinical follow-up in the control vs. intervention group. **A** Thrombembolic and bleeding complications at long-term follow-up. Severe adverse events were rare and without statistically significant difference between the two groups. *TIA* transient ischemic attack. **B** Hospitalization rates during follow-up. Overall hospitalization rates (left columns) and the respective underlying diagnoses leading to hospitalization are shown. Patients in the control group were more often affected by AF-related hospitalization, whereas patients in the intervention group were more often hospitalized for non-cardiac reasons. *AF* atrial fibrillation, *CV* cardiovascular, *FU* follow-up

At follow up, there was no significant difference in stroke, transient ischemic attack, or myocardial infarction between groups (Fig. 3A). Severe bleeding complications were rare events and showed no statistically significant difference between subgroups (Fig. 3A). More moderate bleeding complications were recorded in the intervention group (control group: 2.9%, intervention group: 5.2%; *P* = 0.021). Re-hospitalization rates during follow-up did not differ significantly between the control group and the intervention group (Fig. 3B). However, AF-related re-hospitalization was more common in the control group, whereas non-cardiovascular re-hospitalization rates were elevated in the intervention group (Fig. 3B). As to the number of outpatient medical contacts during the follow-up period, there was no statistical difference between the two groups (control group: 1.8 ± 1.9 visits per quarter of the year; intervention period: 1.8 ± 1.6 visits, *P* = 0.28).

**Fig. 4:**
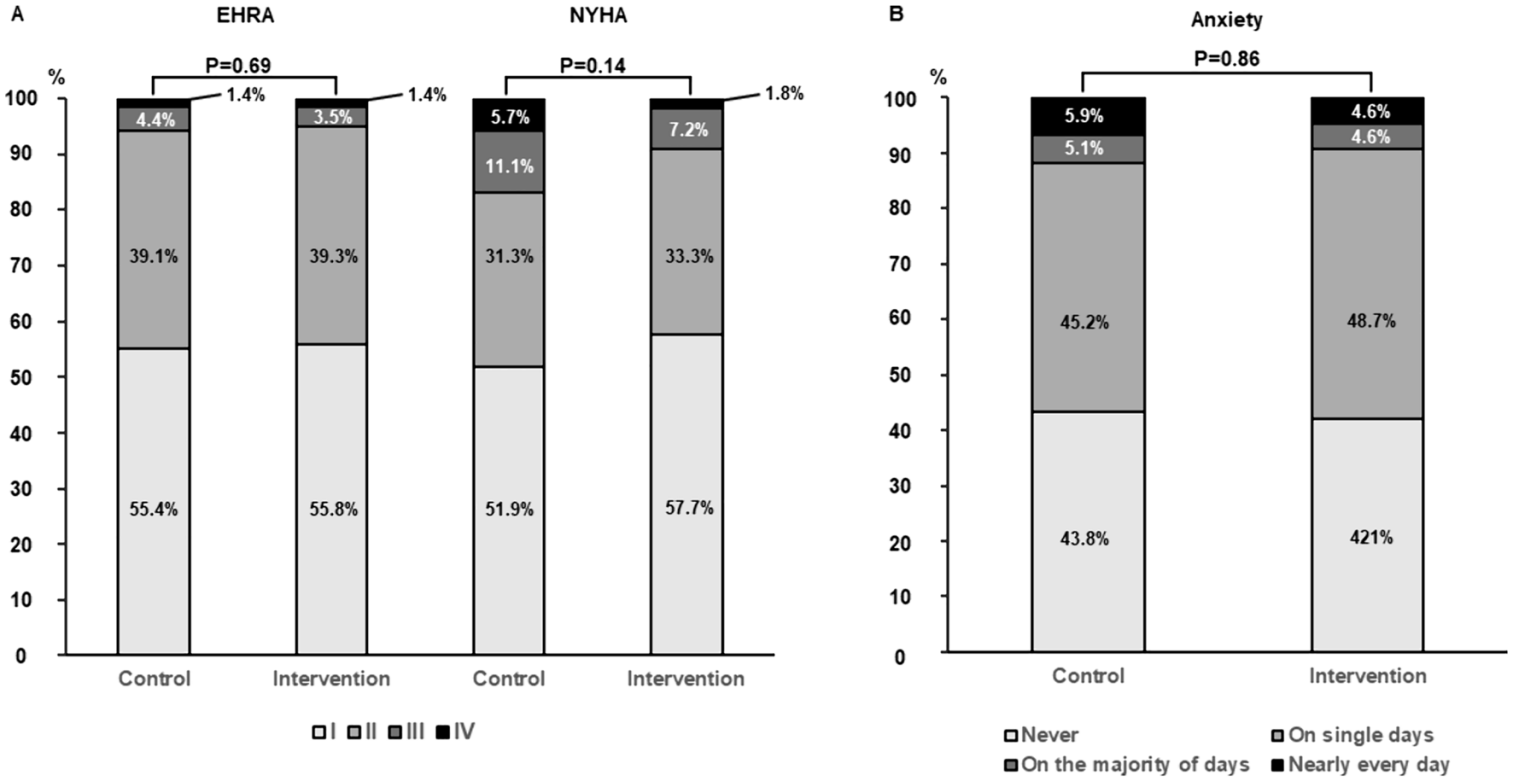
Cardiac symptoms and health-related psychological burden at follow-up. **A** AF-related symptoms characterized by EHRA states (left columns) and physical capacity classified by NYHA states (right column) in the control vs. intervention group at follow-up. Symptoms showed no significant difference between the two groups. *EHRA* European Heart Rhythm Association; *NYHA* New York Heart Association. **B** Patient-reported cardiac anxiety and nervousness in the control vs. intervention group. In both subgroups, comparable levels of anxiety were reported

In the control group, more cardioversions (control group: 9.1%, intervention group: 5.3%, *P* = 0.002) and catheter ablations of AF had been performed during follow-up (control group: 12.0%, intervention group: 5.1%, *P* < 0.001). Regarding the severity of AF-related symptoms, there was no difference between the two groups (EHRA III or IV in the control group: 5.8%, intervention group: 4.9%, *P* = 0.46, Fig. 4A). However, in comparison to baseline, an improvement in AF-related symptoms of survivors reflected in EHRA states could be detected in both groups (signed-rank test *P* < 0.001).

About one in ten patients was member in an AF-related self-help group (control group: 13.1%, intervention group: 11.6%, *P* = 0.39). Quality of life as measured by the EQ-5D-5L-index at long-term follow-up was not different between the two groups at follow-up (control group: 0.91 [Q1 = 078; Q3 = 0.97], intervention group: 0.88 [Q1 = 0.75; Q3 = 0.97], *P* = 0.086). Specific interviews regarding anxiety and cardiophobia revealed no statistically significant difference between groups at follow-up, with a reduction in AF-related anxiety in comparison to baseline interrogation (Supplemental Table S1, Fig. 4B).

When interviewed regarding the role of the ARENA project with respect to the individual health awareness, 24.6% of patients in the control group and 22.2% of patients in the intervention group reported that the ARENA project had influenced their health perception (*P* = 0.27).

### Stroke prevention and medical arrhythmia therapy

At long-term follow-up, more patients in the intervention group received oral anticoagulation therapy according to available prescription regimens (Table 2 and Fig. 1). In comparison to the baseline visit, there was a more pronounced decrease in OAC prescription in the control group in comparison to the intervention group (Table 2). In both groups, the use of VKA decreased in comparison to baseline, and NOACs were the preferred choice for OAC therapy (Table 2 and Fig. 1). Prescription rates of antiplatelet agents decreased in both groups (Table 2).

At follow-up, the rates of beta-blocker prescription showed no significant change in both the control group and the intervention group, and were not different between the two groups (Table 2). Also, rates of antiarrhythmic medical therapy were similar to baseline in the control group and intervention group, and not statistically different between the two groups at follow-up (Table 2).

To exclude patients with a temporary indication for OAC at baseline, e.g., after cardioversion with low CHA_2_DS_2_-VASc score, we performed a subgroup analysis of patients with only class I or class IIa indication for permanent OAC therapy. Data on anticoagulation regimens were available in 718 patients with permanent OAC indication in the control group and in 710 patients in the intervention group. In this subgroup analysis, the rates of oral anticoagulation were not significantly different between the groups at follow-up (control group: 87.5%, intervention group: 90.1%, *P* = 0.11). Monotherapy with antiplatelet agent not in combination with any OAC was present in 3.8% of patients in the control group and 4.8% of patients in the intervention group with a class I/IIa indication for OAC (*P* = 0.36). Rates of severe thromboembolic or bleeding complications in this subgroup analysis during follow-up were comparable to the overall cohort (stroke in the control group: 1.1% vs. intervention group: 1.2%, *P* = 0.94; TIA in the control group: 1.7% vs. intervention group: 1.5%, *P* = 0.75; myocardial infarction in the control group: 0.8% vs. intervention group: 1.6%, *P* = 0.18; severe bleeding complication in the control group: 1.4% vs. intervention group: 2.0%, *P* = 0.38). In analogy to the overall cohort, re-hospitalization rates were similar between the control and intervention group (control group: 50.6%, intervention group: 50.9%, *P* = 0.92), with a higher proportion of AF-related re-hospitalizations in the control group (control group: 31.1%, intervention group: 18.7%, *P* < 0.001).

## Discussion

The ARENA study offers insight into the therapeutic strategies and long-term outcome in a large, regional cohort of patients with AF. High compliance with OAC therapy according to current guideline recommendations was associated with a low rate of severe thromboembolic events and severe bleeding complications requiring medical intervention. Nevertheless, the significant health burden associated with AF was evident in high hospitalization rates and frequent medical contacts. A widespread community-based campaign employing a multi-strategic approach and different public information channels was associated with a higher use of OAC therapy at follow-up.

The ARENA population corresponds to previous large AF cohorts with respect to the baseline parameters. [9, 10] In comparison to the GLORIA-AF phase III registry, globally analyzing the prescription patterns of different OACs, age, co-morbidities, and thromboembolic risk quantified by the CHA_2_DS_2_-VAsc score were similar. [9] The proportion of female patients was lower in the ARENA cohort (37.4% vs. 44.9%). The GLORIA-AF registry already observed a trend toward prescribing NOAC rather than VKA, particularly in newly diagnosed AF. [9, 11] While patients in GLORIA-AF were recruited between 2014 and 2016, ARENA started recruitment in 2016 and continued follow-up until August 2021. The trend toward preferential prescription of NOACs in clinical practice already observed in GLORIA-AF, corroborated by the AF guidelines set forth by the European Society of Cardiology (ESC) from 2016 onward, continued in the ARENA cohort. During the follow-up period in ARENA, the proportion of patients receiving VKA decreased continuously. Factor Xa inhibitors were the preferred choice for NOAC therapy in this cohort. This may be due to the favorably low bleeding and cardiovascular risk reported in large patient cohorts under factor Xa inhibitor therapy in comparison to dabigatran and the high proportion of elderly patients prone to bleeding and cardiovascular complications in the ARENA cohort. [12, 13] Additionally, the number of patients receiving antiplatelet therapy in patients with a class I or IIa indication for OAC was very low during follow-up in ARENA and lower than in the GLORA-AF cohort, which reflects optimized adherence to current guidelines on stroke prevention in AF over the years. [11] Successful implementation of current evidence on stroke prevention and a high rate of adherence to current guidelines was associated with a low rate of serious thromboembolic or bleeding complications.

The ARENA cohort reflects patients affected by AF in a regional, well-balanced population with excellent access to health-care institutions with expertise in AF-therapy. A European patient survey showed relevant disparities regarding knowledge about AF and associated therapies depending on the level of education, relevantly affecting the quality of OAC therapy. [14] With respect to other cardiovascular conditions or risk factors, population-based measures have been associated with improvements in clinical outcome, particularly in underserved communities. [4] In AF, professional education programs on OAC guidelines directed at physicians have been shown to improve patient care with respect to stroke prevention. [15] However, in populations with already high OAC adherence at baseline, widespread campaigns primarily targeting health-care providers have failed to show additional benefit to stroke prevention therapy. [16] In these populations, campaigns integrating patient-directed educational measures were more successful. [17, 18] In the multinational IMProve treatment with AntiCoagulanTs in patients with Atrial Fibrillation (IMPACT-AF) trial, a multifaceted educational intervention directed at both patients and providers was associated with an increase in OAC use. [18] Most studies investigating the effect of educational interventions in AF patients have not yet shown an influence on prognostic endpoints, whereas disease awareness, therapy adherence, and patient competence were influenced in a beneficial manner. [17–22] In the ARENA project, the regionality of the cohort enabled assessment of widespread effects of local structural or demographic developments and public campaigns. In “ARENA intervention”, the effect of a regional information campaign employing multiple methods of public outreach was studied. With respect to stroke prevention therapy, more patients in the intervention group received OAC at long-term follow-up compared to the control group. This may point toward a higher adherence to prescribed medication with improved patient education and awareness for the underlying rationale of recommended therapeutic measures. However, in a subgroup analyses of only patients with class I and IIa indication for long-term OAC therapy, there was no statistically significant difference between the two groups. One reason may be that the intervention group may have included patients who continued OAC therapy despite weak or only temporary indication due to increased awareness of the stroke risk associated with AF. Moreover, the size of the patient cohort in this subgroup analysis may not have allowed for sufficient power to demonstrate the effect of the invention. Additionally, patients in the control group had a lower thromboembolic risk. Thus, this group may have included more patients who only had a temporary indication of OAC therapy, e.g., after cardioversion or catheter ablation.

Higher OAC use in the intervention group was associated with a higher rate of non-severe bleeding events; however, the rate of severe bleeding complications was not statistically significantly increased. At the same time, severe thromboembolic events were low in both groups.

Medical or interventional rhythm control therapy was initiated in a small subgroup of patients of the overall ARENA cohort and a large proportion of patients reported no or only mild symptoms or reduction in quality of life associated with AF at baseline. Symptoms were more pronounced at baseline in patients of the control group who more often received cardioversion and catheter ablation during follow-up. These observations point to the fact that therapy stratification to rhythm control may have been driven by clinical presentation rather than potential prognostic implications in patients with cardiovascular co-morbidities. Prognostic implications in a population at risk were highlighted by the Early Treatment of Atrial Fibrillation for Stroke Prevention Trial (EAST-AF) in 2020, after the conclusion of the ARENA study [23].

The intervention group was characterized by less AF-related anxiety at baseline and less AF-related hospitalizations during follow-up. A more elaborate patient education with the help of community information campaigns may have supported a higher level of patient competence in adhering to and using strategies to avert symptom deterioration requiring hospitalization. However, also in the control group AF-related anxiety was reported less often at follow-up in comparison to baseline. In both subgroups, a quarter of all patients reported that participating in ARENA had influenced their health perception. These observations highlight that the general participation in structured and specialized follow-up programs may be beneficial for selected patients to increase disease awareness and mental well-being.

### Limitations of the study

The ARENA project was a registry study with inherent limitations. The end points were analyzed in a descriptive manner. To reach a broad population of AF patients, a variety of recruitment strategies was used. However, the contribution of recruitment patterns and therefore the availability of baseline information changed over time.

Only five medical practices participated in ARENA despite widespread recruitment efforts via local health organizations and physicians’ professional associations. Due to structural limitations, many practices were not able to provide the additional organizational and personal capacities for research purposes, particularly as ARENA entailed rather comprehensive data collection. It cannot be excluded that the patients included in ARENA do not reflect the entirety of AF patients in the region. However, the ARENA cohort represents a typical AF cohort as reflected by the baseline characteristics and is comparable to previous trials in the context of AF.

During the long-term follow-up period in this large and diverse cohort of patients, a number of patients were lost to follow-up. Follow-up was conducted in a centralized and standardized way and was based on patient-reported outcomes. For defined severe clinical events leading to hospitalization, the respective written medical records were procured to confirm the reported end point, but not adjudicated by a clinical event committee. Follow-up regarding the end point of mortality was completed with information obtained from local registration offices. More specific information enquired in the extensive and comprehensive questionnaire could not be independently confirmed for the entire large cohort of patients and not all pre-defined end points could be completely assessed. We list reasons for incomplete follow-up in “Results” and indicated the number of patients with available data in the respective figures and tables. Importantly, the rate and reasons of loss to follow-up were not statistically different between the two subgroups. Patients’ reply to follow-up contacts by the IHF was delayed in a number of cases, and due to unavailability of personnel the follow-up itself was subject to delays. Consequently, the follow-up periods range widely. Thus, analysis of prognostic end points was restricted to 356 days as event dates were available, whereas the duration of long-term follow-up for other outcomes is indicated accordingly in “Methods” and “Results” of the manuscript. For variables concerning patient status, this variability cannot be corrected in the analysis. With respect to low rates of adverse events, a potential underreporting cannot be excluded.

In the subproject “ARENA intervention” there were statistically significant baseline differences between the two subgroups which have to be taken into consideration when interpreting follow-up results. The follow-up period of most of the control group overlaps with the start of the community-based information campaign. Therefore, the intervention might have influenced the outcomes of the control group and diminished the effects seen in the data. The reasons for discontinuation of OAC were not recorded in the majority of cases. This additionally complicates the assessment of the contribution of the community intervention to OAC adherence. To specify analyses on OAC use in correspondence to the individual risk for thromboembolic complications and current guideline recommendations, subgroup analyses of patients with class I/IIa indication for long-term OAC therapy were performed.

## Conclusion

The ARENA study reflects high adherence to guidelines with respect to stroke prevention in AF in a large, regional cohort, associated with a low rate of serious adverse events. A widespread community-based information campaign was associated with higher adherence to OAC therapy. Structured education programs may facilitate disease awareness and patient self-efficacy in disease and therapy handling, potentially leading to more efficient utilization of health-care providers. The long-term economic effect of patient education on a community level focusing on AF, a disease with significant and increasing health burden in our society, should be further investigated by future studies.

## Supplementary Information

Below is the link to the electronic supplementary material.

Supplementary file1 (DOCX 16 KB)

## Data Availability

The data contributing to this work are available from the corresponding author upon reasonable request, as far as compatible with respective personal data protection regulations.
