# Supplementary material for: Community-based analysis of stroke prevention and effect of public interventions in atrial fibrillation: results from the ARENA project
Source: Clin Res Cardiol. 2024 Aug 8;114(1):138–49. doi: 10.1007/s00392-024-02510-6 (PMC11772519; doi:10.1007/s00392-024-02510-6)
Supplement: Supplementary file 1 — Supplementary file1 (DOCX 16 KB) [file 392_2024_2510_MOESM1_ESM.docx]

**SUPPLEMENTAL MATERIAL**

**Supplemental table S1: Psychosocial evaluation and anxiety at baseline**

|  | **Control baseline (N=973)** | **Intervention baseline (N=927)** | **P-value** | **Control follow-up**  **(N=785)** | **Intervention**  **follow-up (N=786)** | **P-value** |
| --- | --- | --- | --- | --- | --- | --- |
| **Nervousness, anxiety** |  |  | **0.002** |  |  | 0.86 |
| None , (%, N) | 32.6 (297/911) | 39.8 (352/884) |  | 43.8 (344/785) | 42.1 (331/786) |  |
| On single days, (%, N) | 54.3 (495/911) | 49.1 (434/884) |  | 45.2 (355/785) | 48.7 (383/786) |  |
| On the majority of days, (%, N) | 7.1 (65/911) | 6.3 (56/884) |  | 5.1 (40/785) | 4.6 (36/786) |  |
| Nearly every day, (%, N) | 5.9 (54/911) | 4.8 (42/884) |  | 5.9 (46/785) | 4.6 (36/786) |  |
| **Uncontrollable fear** |  |  | 0.67 |  |  | 0.33 |
| None, (%, N) | 50.4 (441/875) | 51.6 (429/831) |  | 57.9 (444/767) | 54.3 (414/763) |  |
| On single days, (%, N) | 40.7 (356/875) | 39.5 (328/831) |  | 33.5 (257/767) | 38.9 (297/763) |  |
| On the majority of days, (%, N) | 5.9 (52/875) | 5.3 (44/831) |  | 5.2 (40/767) | 4.5 (34/763) |  |
| Nearly every day, (%, N) | 3.0 (26/875) | 3.6 (30/831) |  | 3.2 (26/767) | 2.4 (18/763) |  |
| **Fear of acute cardiac event** |  |  | **0.042** |  |  | 0.61 |
| Never, (%, N) | 15.6 (143/916) | 17.1 (152/890) |  | 23.2 (184/793) | 10.7 (164/793) |  |
| Rarely, (%, N) | 29.0 (266/916) | 32.6 (290/890) |  | 32.4 (257/793) | 33.7 (267/793) |  |
| Sometimes, (%, N) | 38.3 (351/916) | 35.4 (315/890) |  | 31.8 (252/793) | 34.7 (275/793) |  |
| Often, (%, N) | 11.8 (108/916) | 10.9 (97/890) |  | 8.8 (70/793) | 8.1 (64/793) |  |
| Constantly, (%, N) | 5.2 (48/916) | 4.0 (36/890) |  | 3.8 (30/793) | 2.9 (23/793) |  |
| **Avoid increasing heart rate** |  |  | **<0.001** |  |  | 0.29 |
| Never, (%, N) | 23.9 (218/911) | 29.1 (259/890) |  | 33.6 (265/788) | 22.8 (268/795) |  |
| Rarely, (%, N) | 26.8 (244/911) | 29.1 (259/890) |  | 24.1 (190/788) | 26.9 (213/793) |  |
| Sometimes, (%, N) | 28.2 (257/911) | 25.8 (230/890) |  | 26.4 (208/788) | 27.0 (214/793) |  |
| Often, (%, N) | 14.3 (130/911) | 11.3 (101/890) |  | 11.5 (91/788) | 8.6 (68/793) |  |
| Constantly, (%, N) | 6.8 (62/911) | 4.6 (41/890) |  | 4.3 (34/788) | 3.8 (30/793) |  |

FU=follow-up
